# Supplementary material for: Objective Estimation of Sensory Thresholds Based on Neurophysiological Parameters
Source: Front Neurosci. 2019 May 16;13:481. doi: 10.3389/fnins.2019.00481 (PMC6532536; doi:10.3389/fnins.2019.00481)
Supplement: Supplementary file 1 [file Data_Sheet_1.PDF]

Supplementary Material
Objective estimation of sensory thresholds based on neurophysiological parameters

Achim Schilling, Richard Gerum, Patrick Krauss, Claus Metzner,
Konstantin Tziridis and Holger Schulze

S1 Generalized logistic function with additive term

Here we show the importance of proper decoupling of the noise and neuronal signal in the mathematical description, and how the naive approach of simply using an additional noise term introduces a measurement repetition dependency in the threshold estimates.

In contrast to the fit function described in the main paper, the data could also be fitted with a generalized logistic function with constant offset as shown in (equ. S1.1) instead of adding the squared noise and calculating the square root (cf. equ. (1)).

$$f(x) = \frac{a}{1 + e^{-\frac{x-b}{c}}} + \sigma \tag{S1.1}$$

Note that the noise amplitude σ is no free parameter to be fitted but set to a constant value extracted from the data as described in the main paper (black dashed line in Suppl. Fig. 1). Trivially a noise dependent criterion (cf. equ. S1.2) such as the 2σ criterion leads to a divergence of the determined threshold as a function of the number of measurement repetitions (cf. Suppl. Fig. 1c) in analogy to Fig. 2B.

$$t_\sigma = -c \cdot \ln\left(\frac{a}{\sigma} - 1\right) + b \tag{S1.2}$$

However, it can be shown that even the 5 % criterion leads to a monotonic decrease of the determined threshold as a function of the applied measurement repetitions (i.e. the sample size, cf. Suppl. Fig. 1). Nevertheless, it has to be considered that in contrast to the 2σ criterion the threshold as a function of the number of measurement repetitions saturates at a certain value and does not diverge. Thus,

the threshold for a real measurement is systematically overestimated compared to the case of no measurement noise. This limitation can be overcome by using the fit function described in the main paper (cf. equ. (1)).

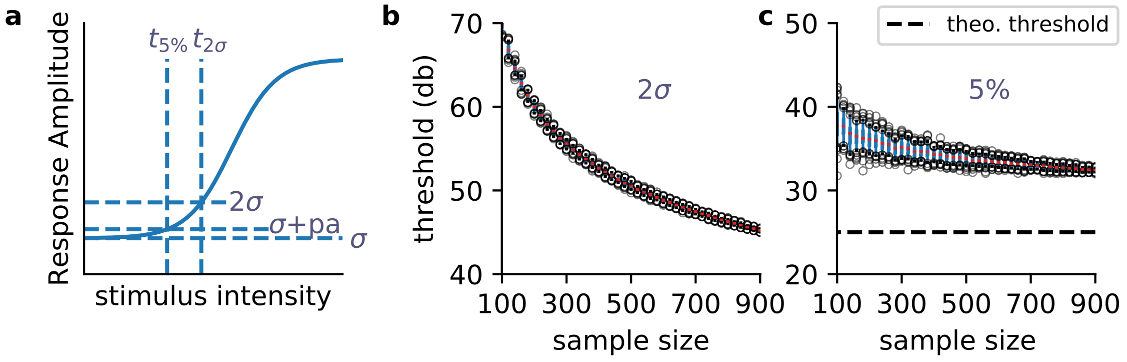

Suppl. Fig. 1 | Schematic representation of the fitted generalized logistic function. **a**, Schematic generalized logistic function which is fitted to the RMS values of the ABR Waves. σ marks the amplitude of the background noise. The parameter σ is no fit parameter but is measured under non-stimulus condition and kept constant to reduce the number of variable parameters. The other two (blue dashed) horizontal lines mark different threshold criteria (fraction criterion: $\sigma + p \cdot a$ and the background noise dependent criterion 2σ-criterion. The threshold is set to the value where the fitted function exceeds the horizontal lines. **b**, **c**, Determined threshold as a function of the number of applied measurement repetitions; the background noise criterion (**b**) and for the fraction criterion (p-criterion) (**c**). The theoretical threshold for the p=5% criterion is set to 25 dB (artificial data). The theoretical threshold of the 2σ-criterion is $-\infty$, meaning that the function diverges for lower measurement noise and a higher number of measurement repetitions.

S2 Derivation of the correct fit function for RMS values

As shown above the generalized logistic function with an additional term σ leads to a direct dependency of the determined sensory threshold on the number of applied measurement repetitions. In the following, we show how the noise contributes to the measured stimulus response function in the case of an RMS value. We consider the calculation of the RMS over time (t_n) where V_{t_n} are the measured values at the point in time t_n (cf. equ. S2.1, $t_M = 10\,\mathrm{ms}$).

$$\mathrm{RMS}(x) = \sqrt{\frac{1}{M}\sum_{n=0}^{M} V_{t_n}^2} = \tag{S2.1}$$

$$\sqrt{\frac{1}{M}\sum_{n=0}^{M} [P_{t_n}(x) + G_{t_n}]^2} = \tag{S2.2}$$

$$\sqrt{\frac{1}{M}\sum_{n=0}^{M} [P_{t_n}(x)^2 + 2P_{t_n}(x)G_{t_n} + G_{t_n}^2]} = \tag{S2.3}$$

$$\sqrt{\frac{1}{M}\sum_{n=0}^{M} P_{t_n}(x)^2 + \frac{1}{M}\sum_{n=0}^{M} 2P_{t_n}(x)G_{t_n} + \frac{1}{M}\sum_{n=0}^{M} G_{t_n}^2} \approx \tag{S2.4}$$

$$\sqrt{\frac{1}{M}\sum_{n=0}^{M} P_{t_n}(x)^2 + \frac{1}{M}\sum_{n=0}^{M} G_{t_n}^2} = \tag{S2.5}$$

$$\sqrt{f_0(x)^2 + \sigma^2} \tag{S2.6}$$

This values are a superposition of evoked response $P_{t_n}(x)$ (x: stimulus intensity) and background noise G_{t_n} (cf. equ. S2.2), $f_0(x)$ represents the RMS values of the pure evoked response and σ the background noise. Squaring the sum generates a mixed term ($2P_{t_n}(x)G_{t_n}$, cf. equ. S2.3) which can be considered to be very small as the noise is assumed to be Gaussian distributed with a mean equal to 0. If the mixed term is neglected, the responses as a function of the sound pressure level x is of the form shown in equ. S2.6. This derivation is valid for the assumption that there is no covariance between background noise and evoked responses.

S3 Derivation of the slope of the extended hard sigmoid fit at the threshold

The fit function used to estimate the RMS values of neurophysiological data as a function of stimulus intensity is the square root of the squared hard sigmoid function and the squared background noise amplitude (cf. equ 2b). The hard sigmoid function shows a clear knee used to define the threshold. However, the fit function itself has no knee but is smoothed directly at the threshold value. For simplification of the derivation the threshold t (knee of the base function) is set to zero.

$$f_0(x) = \begin{cases} 0 & x < 0 \\ s \cdot x & 0 \leq x \end{cases} \tag{S3.1}$$

$$f(x) = \sqrt{f_0(x)^2 + \sigma} \tag{S3.2}$$

$$\lim_{\substack{x \to 0}}^{x>0} f'(x, t = 0) = \frac{d}{dx}\sqrt{(s \cdot x)^2 + \sigma^2} = \frac{sx_0}{\sqrt{(sx_0)^2 + \sigma^2}} = 0 \tag{S3.3}$$

$$\lim_{\substack{x \to 0}}^{x<0} f'(x, t = 0) = \frac{d}{dx}\sqrt{0^2 + \sigma^2} = 0 \tag{S3.4}$$

The slope of the fit function calculated for $x < 0$ equals the slope of the fit function for $x > 0$. Thus, the fit function is differentiable, although the underlying function has a knee.

S4 Effect of fitting the background noise level

In the main text we describe that the noise amplitude σ is not treated as fit parameter but as constant value measured under non-stimulus condition. This methodological principle is of special importance if no supporting points are measured near the threshold (meaning low intensities). If σ is treated as fit parameter (cf. Suppl. Fig. 2) missing supporting points cause unreliable fitting. The robustness of the method can be quantified by the analysis of the determined threshold as a function of the number of deleted supporting points (in analogy to Fig. 3). The deletion of the supporting points leads to a drop in the determined threshold (cf. Suppl. Fig. 2c) for variable noise amplitude (red curve) and stays constant for a fixed noise amplitude (blue curve).

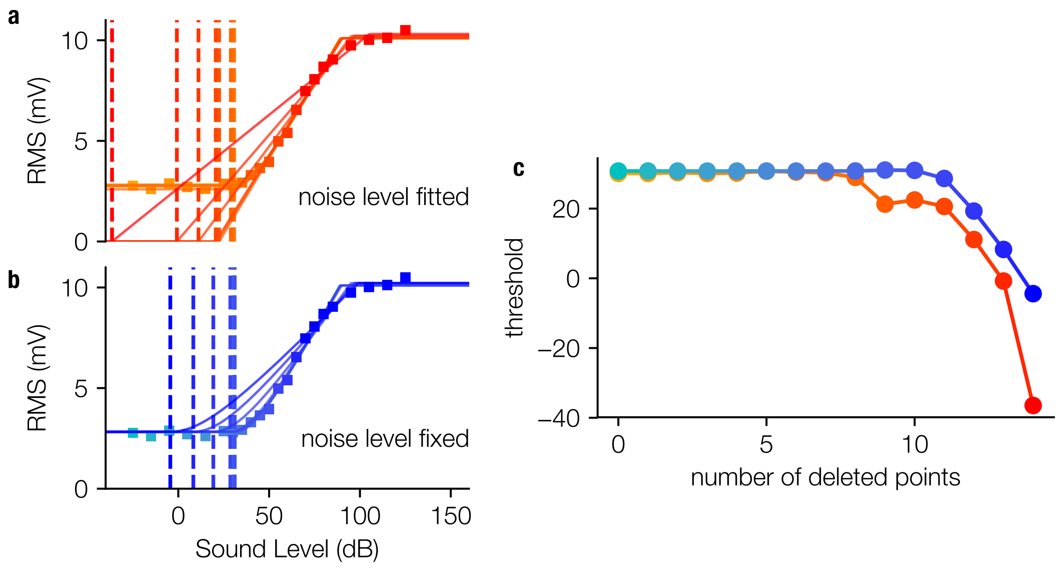

Suppl. Fig. 2 | Stability of threshold estimate with fitted vs fixed background noise level. a, Fit function of a generalized hard sigmoid function with different number of supporting points with the background noise level as a free fitting parameter. **b**, Fit function where the background noise level has been fixed. **c**, Dependence of the estimated threshold value on the number of supporting points. The threshold for the fits with a fixed background noise value (blue) are more stable than the fits with the background noise level as a free parameter (red).

S5 Effect of missing supporting points (real ABR data)

As described in section (Effect of Removal of Supporting Points) fitting of the extended generalized logistic function becomes unreliable for a lack of measured supporting points (cf. Fig. 3d for simulated data) in the saturation range, i.e. for stimuli of high intensity. The analysis of ABR data (Suppl. Fig. 3) illustrates the effect of missing supporting points. As the function is symmetric around the inflection point, the curvature near the threshold depends on the curvature in the saturation range. This results in a coupling of the estimated threshold with the function's shape in the saturation range. Thus, missing supporting points in the saturation often overestimate the fit parameter a and, therefore, can influence the estimated threshold, rendering the threshold estimation unstable (cf. Suppl. Fig. 3d,c).

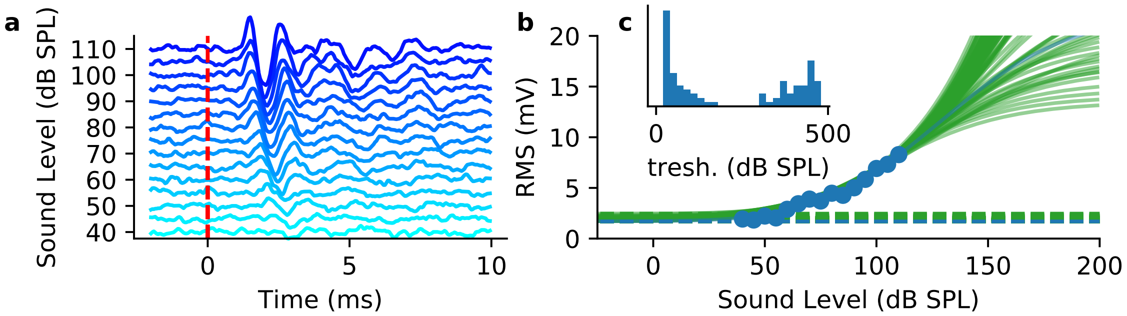

Suppl. Fig. 3 | Unreliable fitting for missing supporting points in the saturation range. a, ABR waves (4 kHz, N=120 double trials, 40-120 dB SPL) measured in a Mongolian gerbil, **b**, Extended generalized logistic fit (in analogy to simulated data cf. Fig. 3d) to the data (green curves: 100 subsamples N=100, blue curve: complete data set). The shape of fit functions (green) highly depends on the used subsample and thus is an unreliable description of the data. Histogram of resulting thresholds; **c** The according threshold estimates scatter over a range of ∼500 dB showing that the fit of the extended generalized logistic function is not robust for missing supporting points in the saturation range
